# Supplementary material for: Generation of functional posterior spinal motor neurons from hPSCs-derived human spinal cord neural progenitor cells
Source: Cell Regen. 2023 Mar 23;12:15. doi: 10.1186/s13619-023-00159-6 (PMC10033800; doi:10.1186/s13619-023-00159-6)

Figure S1. (Related to Figure 1)

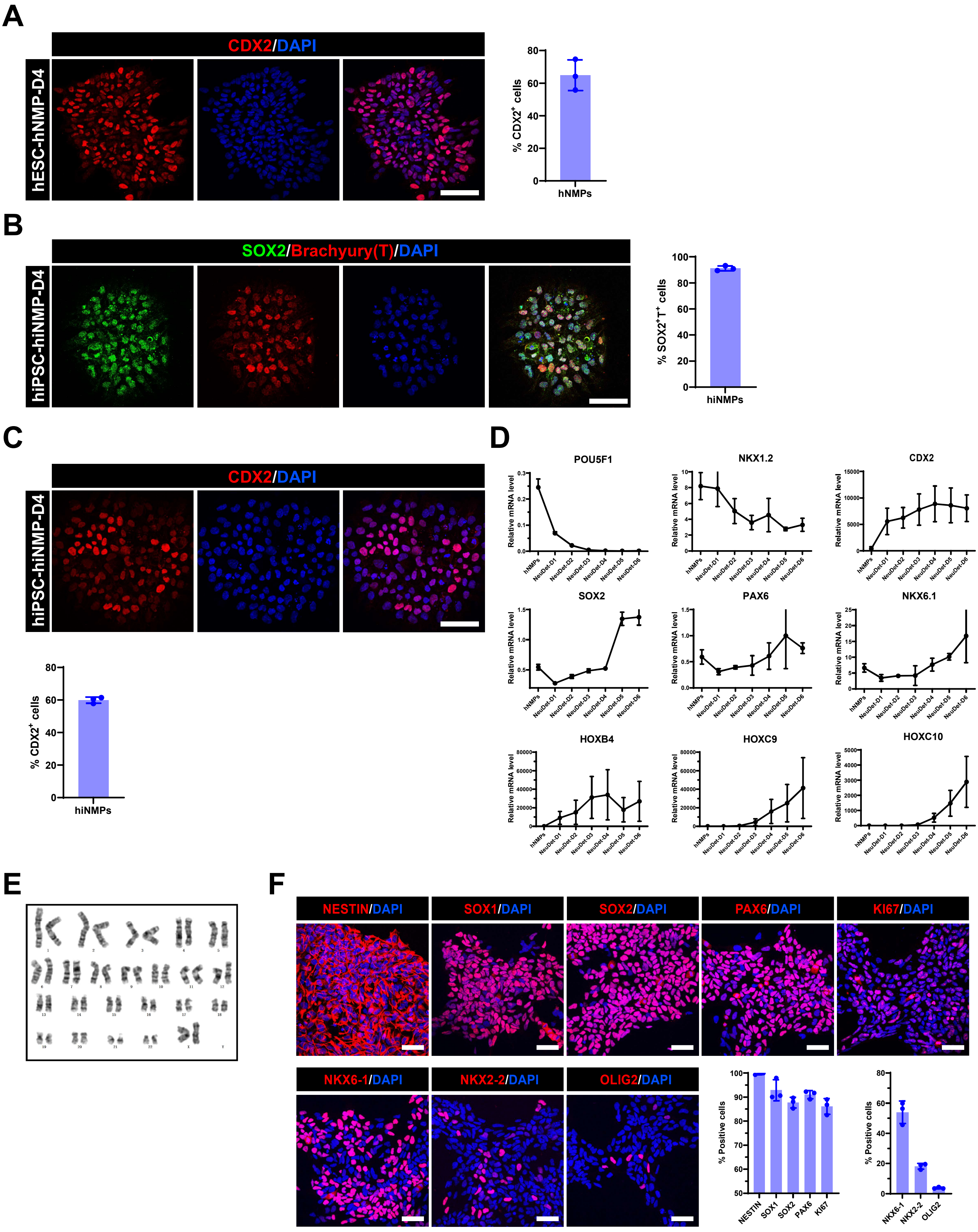

Figure S2. (Related to Figure 2)

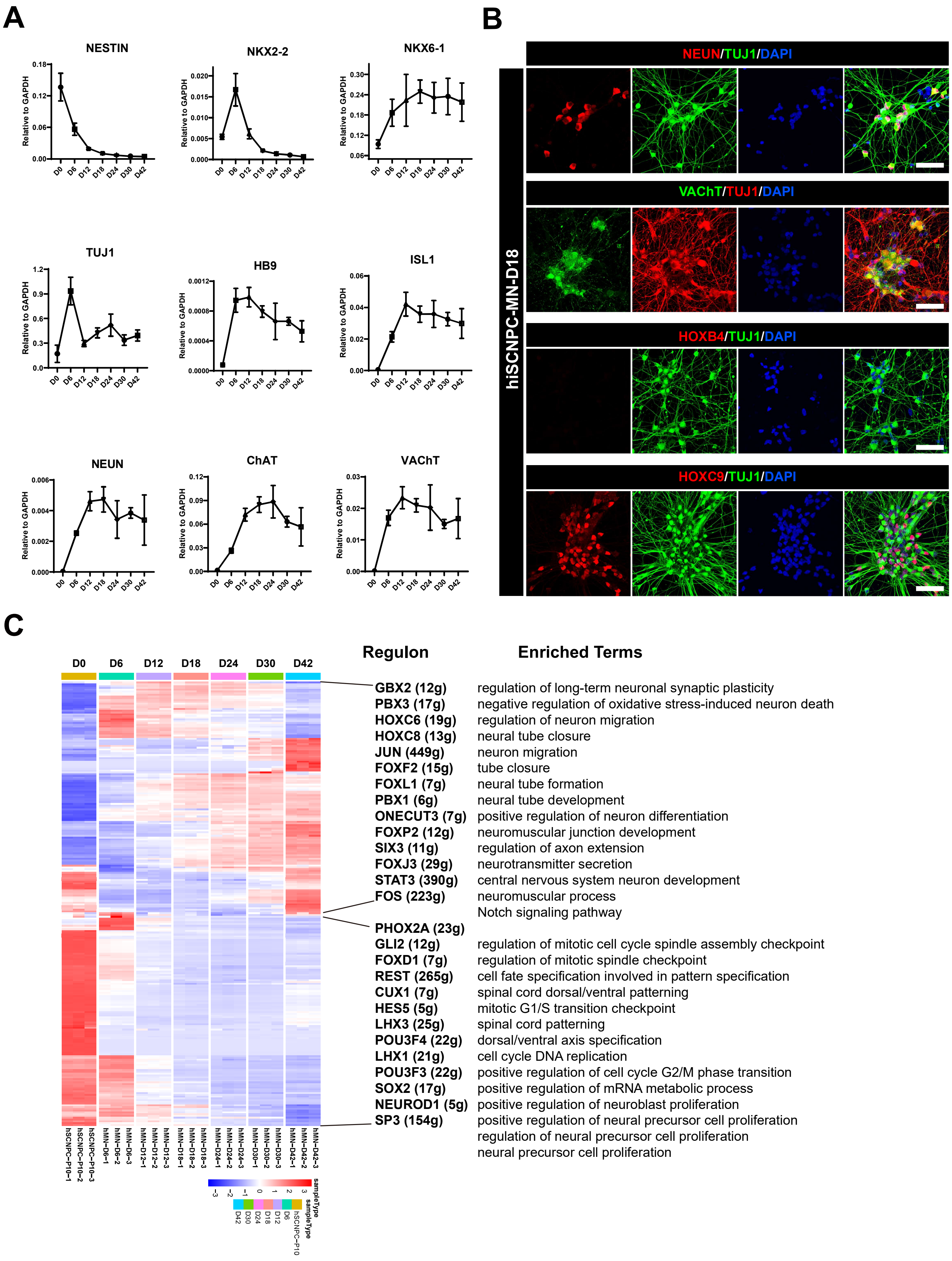

Figure S3. (Related to Figure 2)

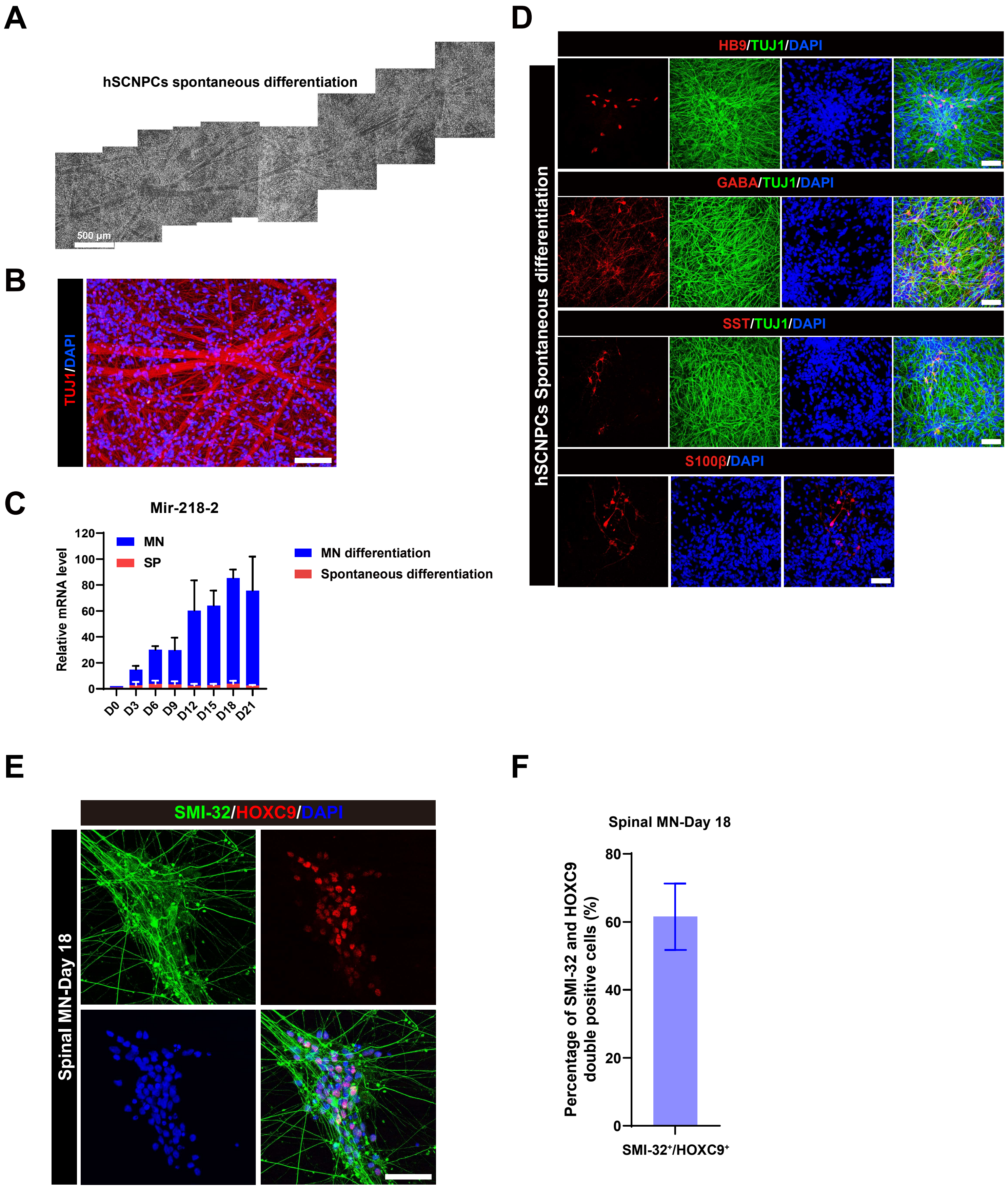

Figure S4. (Related to Figure 2)

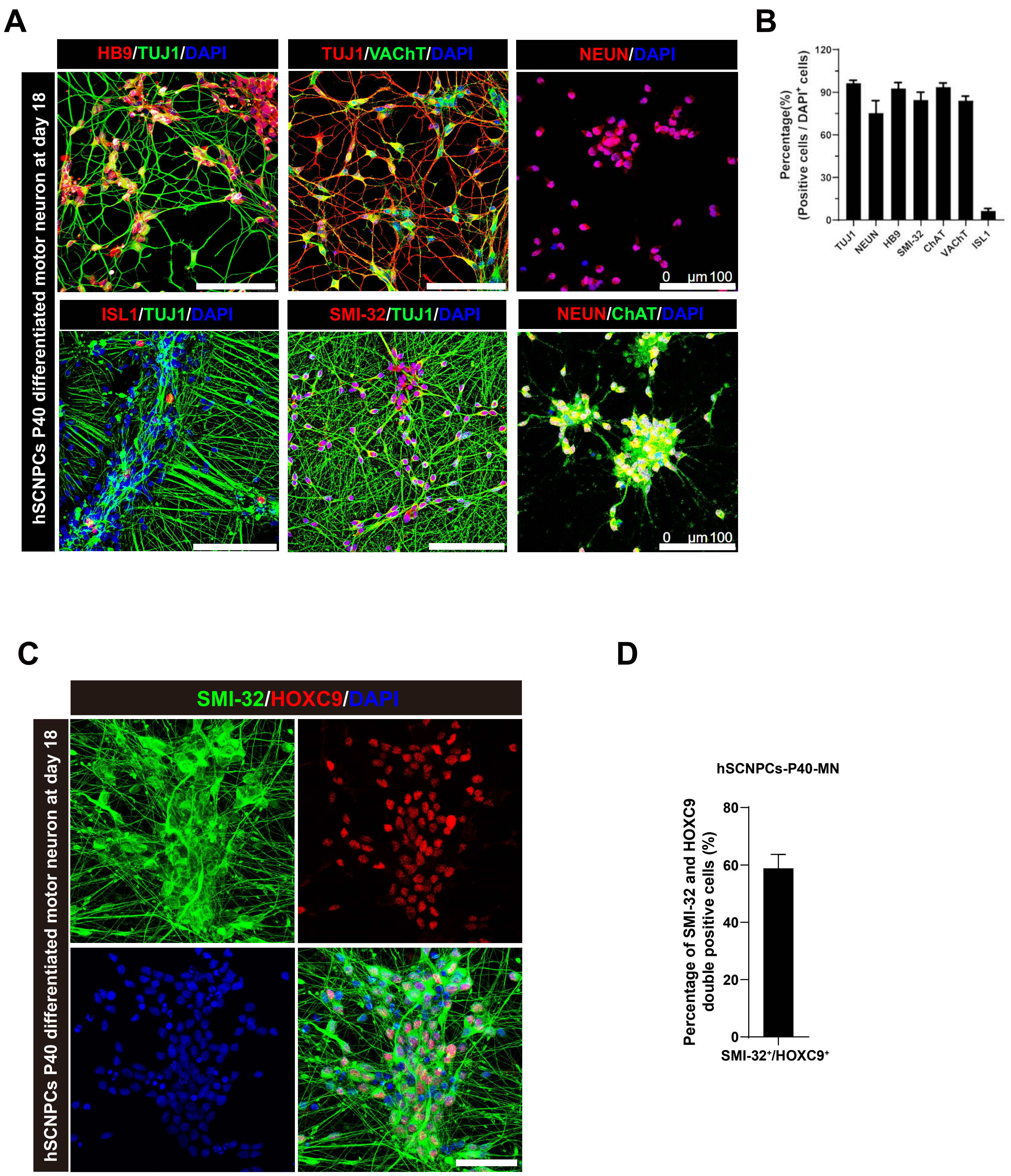

Figure S5. (Related to Figure 3)

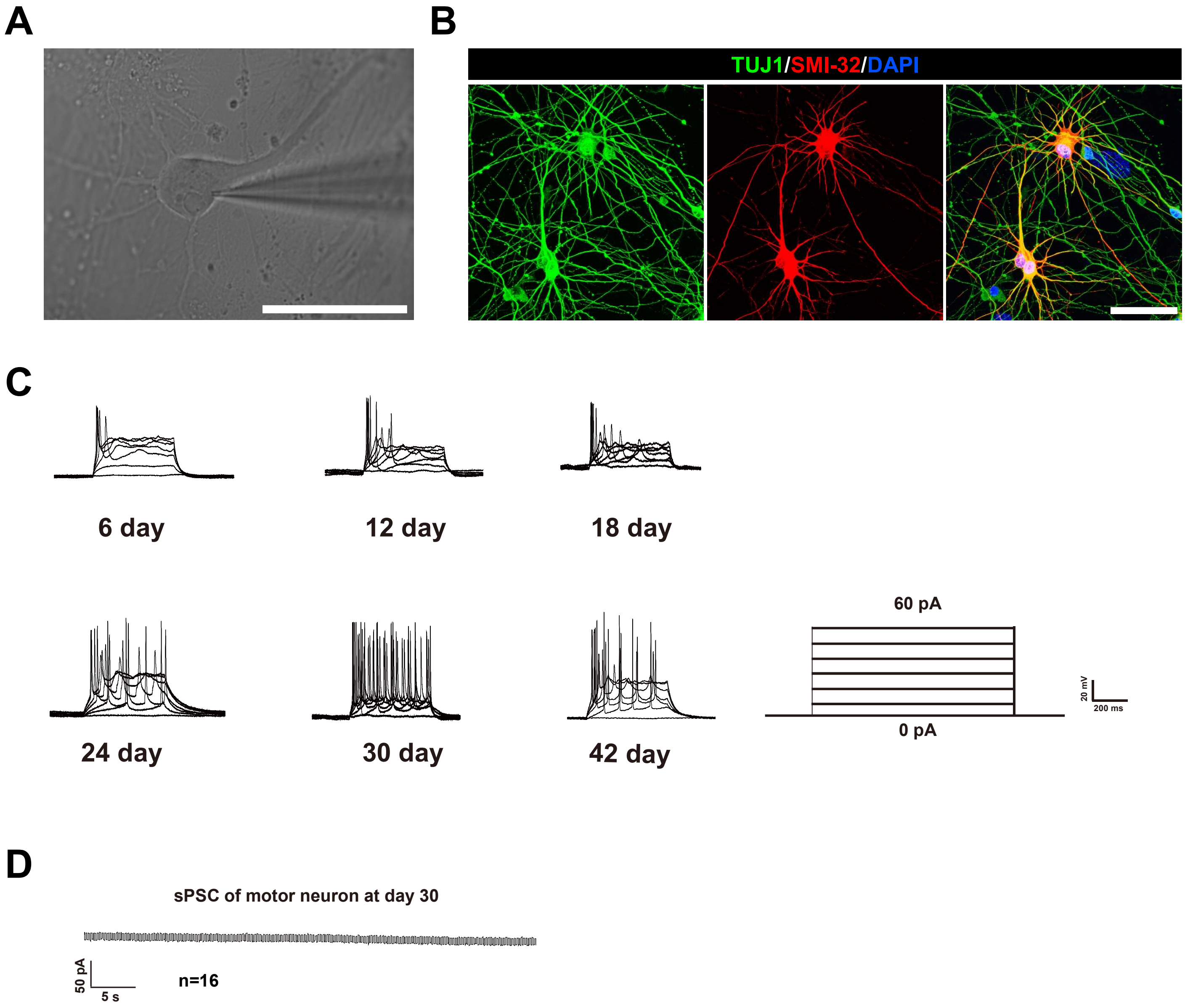

Figure S6. (Related to Figure 5)

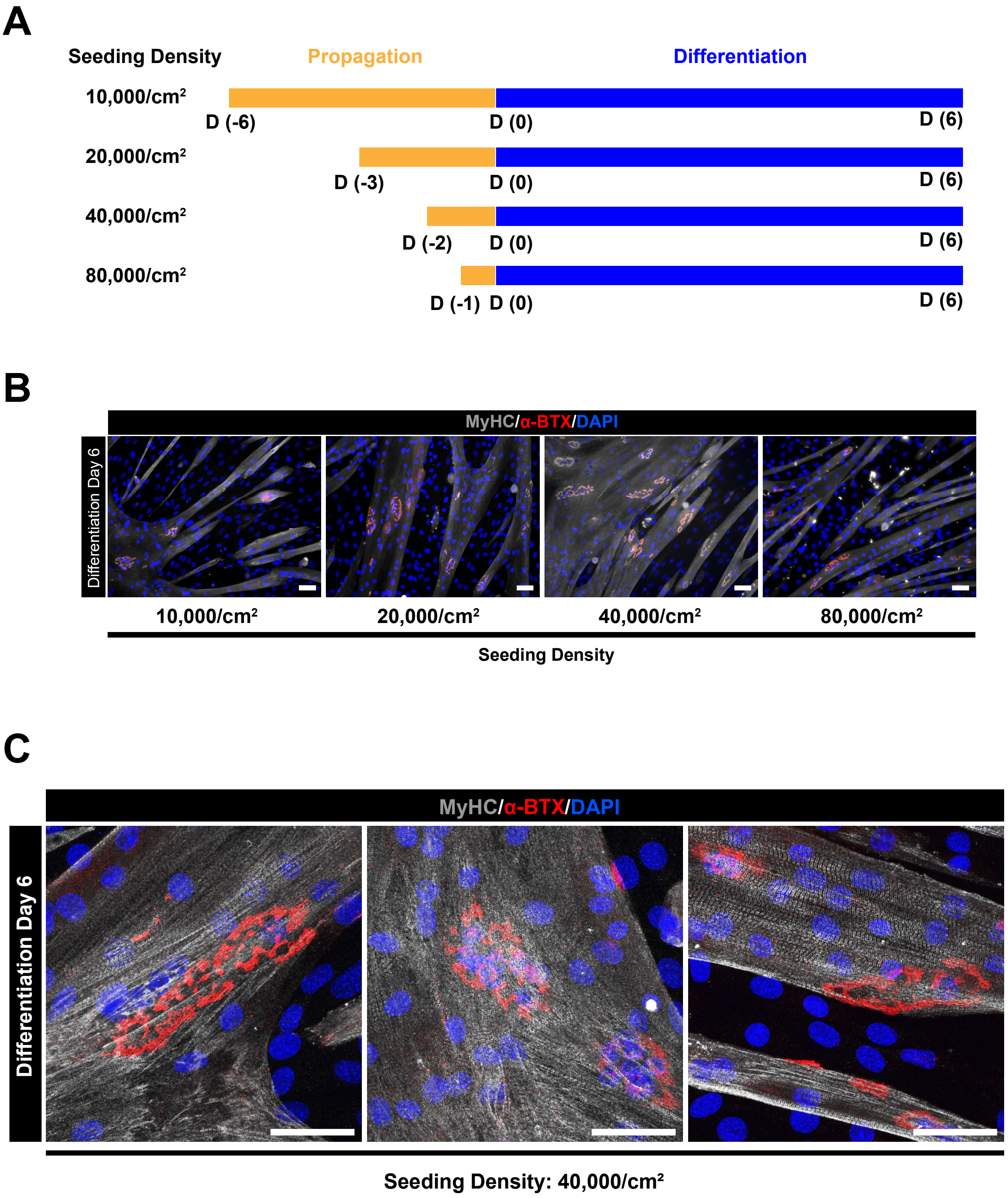

Supplement: Supplementary file 1 — Additional file 1: Fig. S1. Characterization of hNMPs and hSCNPCs from hPSCs. (A) Immunofluorescent staining of hNMP marker (CDX2) and the quantification results. Scale bar, 50 μm. n = 3 independent experiments. Data are represented as mean ± SD. (B) Immunofluorescent staining of hNMP markers (SOX2 and Brachyury) of hiPSCs (DC60-3 and DC87-3)-derived NMPs and the quantification results. Scale bar, 50 μm. n = 3 independent experiments. Data are represented as mean ± SD. (C) Immunofluorescent staining of hNMP marker (CDX2) of hiPSCs (DC60-3 and DC87-3)-derived NMPs and the quantification results. Scale bar, 50 μm. n = 3 independent experiments. Data are represented as mean ± SD. (D) Relative marker genes expression of during NeuDet stage. n = 3 independent experiments. Data are represented as mean ± SD. (E) Karyotyping of hSCNPCs at P40. (F) Immunofluorescence analysis of hiPSCs (DC60-3 and DC87-3)-derived hiSCNPCs and the quantification results. Scale bar, 50 μm. n = 3 independent experiments. Data are represented as mean ± SD. Fig. S2. RNA expression pattern during spinal motor neuron differentiation and characterization of hiSCNPCs-derived spinal motor neurons. (A) Relative marker genes expression during spinal motor neuron differentiation from hSCNPCs. n = 3 independent experiments. Data are represented as mean ± SD. (B) Immunostaining characterization of hiSCNPCs (Derived from hiPSCs lines DC60-3 and DC87-3) differentiated spinal motor neurons at day 18. Scale bar, 50 μm. (C) The heat-map showing two regulon groups in samples of spinal motor neuron differentiation from hSCNPCs with listing representative regulon transcription factors (numbers of predicted target genes by SCENIC in the brackets) and enriched GO terms for each regulon group. Fig. S3. Multipotency of hSCNPCs and efficiency of hSCNPCs differentiate into posterior spinal motor neurons. (A) Bright field of hSCNPCs spontaneous differentiated neurons. Scale bar, 500 μm. (B) Immunofluorescent staining of [file 13619_2023_159_MOESM1_ESM.pdf]
