# Supplementary material for: Generation of functional posterior spinal motor neurons from hPSCs-derived human spinal cord neural progenitor cells
Source: Cell Regen. 2023 Mar 23;12:15. doi: 10.1186/s13619-023-00159-6 (PMC10033800; doi:10.1186/s13619-023-00159-6)
Supplement: Supplementary file 2 — Additional file 2: Table S1. Methods for the identification of HOX genes expression in human NMP-derived neuronal cells. [file 13619_2023_159_MOESM2_ESM.pdf]

Table S1. Methods for the identification of HOX genes expression in human NMP-derived neuronal cells

HOX Genes	*Hiromi Kumamaru et al. 2018*		*Matt Wind et al. 2021*		*Fay Cooper et al. 2022*		*Zachary Olmsted et al. 2022*		*This study*	
	RT-qPCR	ICC	RT-qPCR	ICC	RT-qPCR	ICC	RT-qPCR	ICC	RT-qPCR	ICC
HOX1										
HOX2										
HOX3										
HOX4	+								+	+ (7.2%)
HOX5										
HOX6	+		+		+			+		+ (0%)
HOX7										
HOX8	+		+		+					
HOX9	+		+	+					+	+ (64%)
HOX10	+				+				+	+ (0%)
HOX11										+ (30%)
HOX12										
HOX13					+					

\+ method was used in the identification of relative HOX expression
(%) the percentage of HOX protein expression efficiency
