# Supplementary material for: Generation of functional posterior spinal motor neurons from hPSCs-derived human spinal cord neural progenitor cells
Source: Cell Regen. 2023 Mar 23;12:15. doi: 10.1186/s13619-023-00159-6 (PMC10033800; doi:10.1186/s13619-023-00159-6)
Supplement: Supplementary file 3 — Additional file 3: Table S2. Oligonucleotide sequences, related to STAR Methods. [file 13619_2023_159_MOESM3_ESM.pdf]

Table S1. Oligonucleotide sequences, related to STAR Methods.

RT-qPCR primers	Forward	Reverse
GAPDH	GAGCACAAGAGGAAGAGAGAGACCC	GTTGAGCACAGGGTACTTTATTGATGGTACATG
POU5F1	CGTGAAGCTGGAGAAGGAGAAGCTG	CAAGGGCCGCAGCTTACACATGTTC
NKX1-2	CCCTCCCACCACAAGATTTCT	GACCTCCGCCAAACTTTTCCT
CDX2	GACGTGAGCATGTACCCTAGC	GCGTAGCCATTCCAGTCCT
SOX2	TGGACAGTTACGCGCACAT	CGAGTAGGACATGCTGTAGGT
PAX6	TGGGCAGGTATTACGAGACTG	ACTCCCGCTTATACTGGGCTA
HOXB4	CTGGATGCGCAAAGTTCACGTG	CGTGTCAGGTAGCGGTTGTAGT
HOXC9	CAGCAAGCACAAAGAGGAGAAGG	AGTTCCAGCGTCTGGTACTTGG
HOXC10	GAGCGAAAAGGAGAGGGCCAAA	TCCGCTCTTTGCTGTCAGCCAA
NESTIN	GGCGCACCTCAAGATGTCC	CTTGGGGTCCTGAAAGCTG
NKX2-2	CCTTCTACGACAGCAGCGACAA	ACTTGGAGCTTGAGTCCTGAGG
NKX6-1	CCTATTCGTTGGGGATGACAGAG	TCTGTCTCCGAGTCCTGCTTCT
HB9	GATGCCCGACTTCAACTCCC	GCCGCGACAGGTACTTGTT
ISL1	TGAAATGTGCGGAGTGTAATCAGTATTTGGAC	CACACAGCGGAAACACTCGATGTG
TUJ1	GAGCGGATCAGCGTCTACTAC	CCCCACTCTGACCAAAGATGAA
NEUN	CCAAGCGGCTACACGTCTC	CGTCCCATTCAGCTTCTCCC
VAChT	TTCGCCTCTACAGTCCTGTTC	GCTCCTCCGGGTACTTATCG
ChAT	CATGAAGCAATACTATGGGCTCTTCTCCTC	GACGGCGGAAATTAATGACAACATCCAAG
Mir-218-2	TGCGGGGCTTTCCTTTGT	CCGTTTCCATCGTTCCAC
gapdh	TGTGATGGGTGTGAACCACGAGAA	CTGTGGTCATGAGCCCTTCCACAA
musk	CTGAAGGCTGTGAGTCCACTGT	TCCTTTACCGCCAGGCAGTACT
chrng	CTTGTGGCTAAGAAGGTGCCTG	GCAAGGACACATTGAGCACGAC
chrne	AGACCTGAGGACACTGTCACCA	TCGTCCTTGCTGTAGTTGAGCC
